# Occurrence and genetic characteristics of *Giardia duodenalis* in donkeys in Xinjiang, China[Fn FN1]

**DOI:** 10.1051/parasite/2023052

**Published:** 2023-11-28

**Authors:** Chunyan Xu, Haixin Tuo, Wen Wang, Zhenjie Zhang, Fuchang Yu, Liwen Chuai, Meng Qi, Bo Jing

**Affiliations:** 1 College of Animal Science and Technology, Tarim University, Alar Xinjiang 843300 China

**Keywords:** *Giardia duodenalis*, Infection rate, Genetic characterization, Donkeys

## Abstract

*Giardia duodenalis* is a common enteric parasite in humans and animals. To examine the occurrence and genetic characteristics of *Giardia* in donkeys in Xinjiang, China, 758 fecal samples from donkeys were collected, and *Giardia* was screened via PCR at the *SSU* rRNA gene. A total of 17.0% (129/758) of samples tested positive for *Giardia*, with the infection rate in large-scale farm and domestic donkeys being 21.4% (124/580) and 2.8% (5/178), respectively; the infection rates in <1-year-old and ≥1-year-old donkeys were 19.3% (72/374) and 12.7% (41/323), respectively. Three *Giardia* assemblages were identified, with assemblage B (*n* = 102) as the prevalent assemblage, followed by assemblage A (*n* = 23) and assemblage E (*n* = 4). Of the 129 *Giardia*-positive isolates, 40, 34 and 59 sequences were obtained at the *bg*, *gdh* and *tpi* genes, respectively. Twenty-one isolates successfully allowed multilocus genotyping (MLG), with four novel assemblage A MLGs, named MLG-AI-1 (*n* = 1), MLG-AI-2 (*n* = 1), MLG-AI-3 (*n* = 1), and MLG-AI-4 (*n* = 1) and three novel assemblage B MLGs, named MLG-B1 (*n* = 1), MLG-B2 (*n* = 14), and MLG-B3 (*n* = 1). Moreover, two isolates formed two MLG-mixed sequences. The results suggest that donkeys are commonly infected with *Giardia* in Xinjiang, and there is genetic diversity and host adaptability among the isolates.

## Abstract (fr)

*Giardia duodenalis* est un parasite entérique courant chez les humains et les animaux. Pour étudier la présence et les caractéristiques génétiques de *Giardia* chez les ânes du Xinjiang, en Chine, 758 échantillons fécaux d’ânes ont été collectés et *Giardia* a été criblé par PCR du gène de l’ARNr SSU. Au total, 17,0 % (129/758) des échantillons ont été testés positifs pour *Giardia*. Le taux d’infection, respectivement chez les ânes des élevages à grande échelle et domestiques, étaient de 21,4 % (124/580) et 2,8 % (5/178). Les taux chez les ânes de < 1 an et ≥ 1 an étaient respectivement de 19,3 % (72/374) et 12,7 % (41/323). Trois assemblages de *Giardia* ont été identifiés, l’assemblage B (*n* = 102) étant l’assemblage prédominant, suivi de l’assemblage A (*n* = 23) et de l’assemblage E (*n* = 4). Sur les 129 isolats positifs pour *Giardia*, 40, 34 et 59 séquences ont été obtenues respectivement au niveau des gènes *bg*, *gdh* et *tpi*. Vingt et un isolats ont permis du génotypage multilocus (MLG), avec quatre nouveaux MLG de l’assemblage A, nommés MLG-AI-1 (*n* = 1), MLG-AI-2 (*n* = 1), MLG-AI-3 (*n* = 1) et MLG-AI-4 (*n* = 1) et trois nouveaux MLG de l’assemblage B, nommés MLG-B1 (*n* = 1), MLG-B2 (*n* = 14) et MLG-B3 (*n* = 1). De plus, deux isolats formaient deux séquences MLG mélangés. Les résultats suggèrent que les ânes sont couramment infectés par *Giardia* au Xinjiang, et qu’il existe une diversité génétique et une adaptabilité à l’hôte parmi les isolats.

## Introduction

*Giardia duodenalis* is a common enteric parasite that infects various vertebrates worldwide [6]. Infections can result in clinical symptoms in humans and animals, including profuse and fatty diarrhea, abdominal cramps, nausea, and wasting, representing a serious threat to human health and significant economic losses to farms [8, 14].

Based on genetic sequences of *Giardia* at small subunit rRNA (*SSU* rRNA), eight assemblages (A–H) have been identified, and assemblages A and B were demonstrated to have zoonotic potential [15]. In recent years, multilocus genotyping (MLG) has increasingly been used to genetically characterize *Giardia* isolates from humans and animals. A previous study showed that the MLG of *Giardia* could improve the assignment of each isolate to a specific assemblage and thereby better clarify the transmission and prevalence of giardiasis [2].

Donkeys have a long history as working animals, and they also have high livestock and medicinal value [10]. Previous research conducted in partial areas of China and Iran showed that the range of *Giardia* infection rates was 0%–18.3% [16, 20], and the prevalent assemblages were assemblages A and B. Globally, China has the largest number of donkeys (11 million), with donkey meat, skin, and milk products present ubiquitous in people’s lives. To our knowledge, only two reports about *Giardia* detection in donkeys in China have been published, and more information on *Giardia* in donkeys needs to be uncovered [11, 24]. In this study, the aim was to understand the occurrence and genetic characteristics of *Giardia* in donkeys in Xinjiang, China.

## Materials and methods

### Ethical standards

The protocol in this study was not required to be reviewed and approved by an Animal Studies Ethics Committee. Appropriate permission from farmers or owners was obtained before collecting fecal samples of donkeys, and no animals were harmed.

### Sample collection

**Figure 1:**
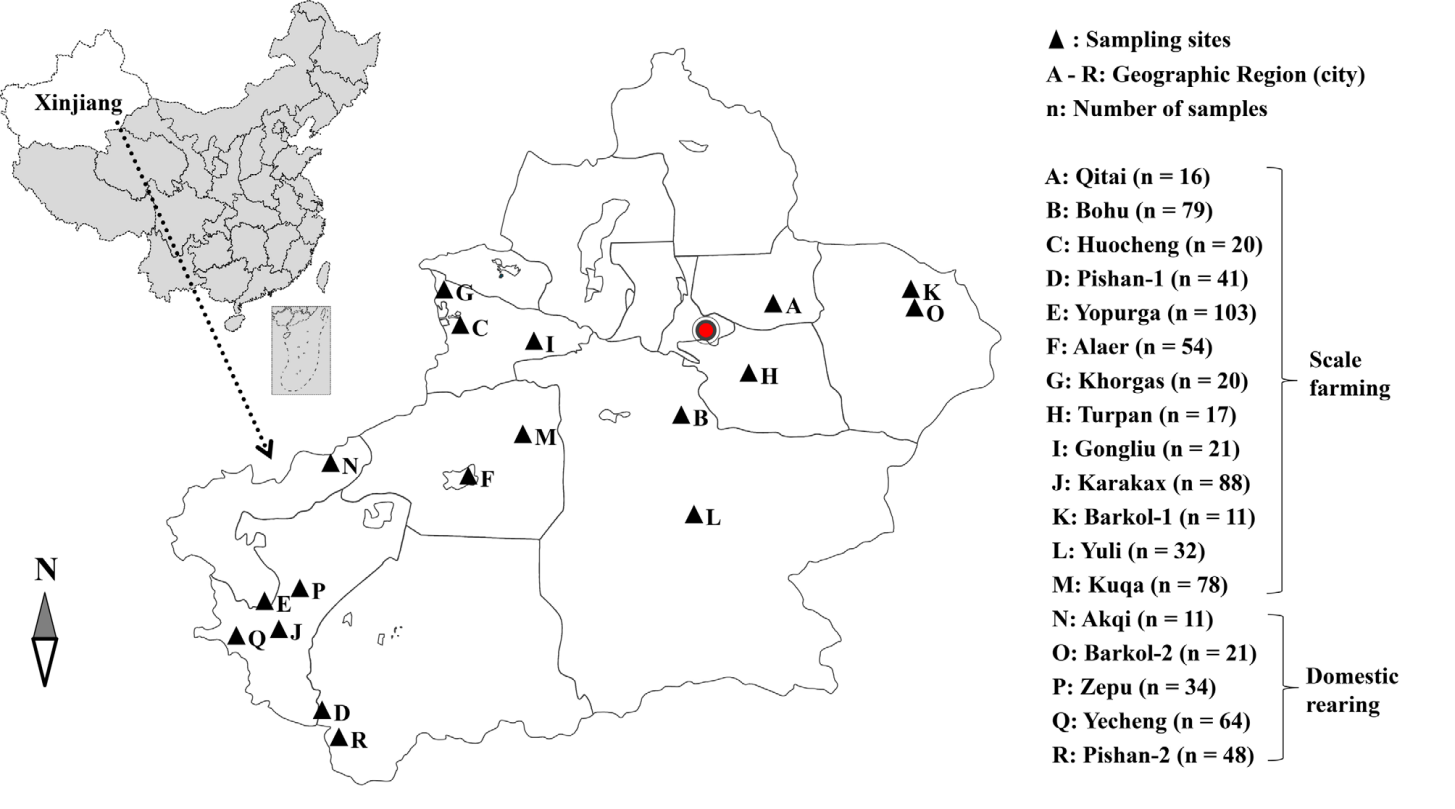
Distribution of sampling locations in southern Xinjiang, China. Filled triangles indicate sampling farms. The letters A–R represent the cities where the sampling sites were located.

From July 2016 to April 2021, 758 fresh fecal samples were collected from 580 farm-raised donkeys in 13 cities and 178 free-ranging donkeys in five countryside areas of Xinjiang, China (Fig. 1). Of these, 374 samples were from young donkeys (<1 year), 323 samples were from adult donkeys (≥1 year), and 61 samples were of undetermined age. All samples were collected when the donkeys defecated, in a sterile disposable latex glove and were placed in labeled sterile bags, transported and stored at 4 °C. No clinical symptoms were observed in any animal at the time of sampling.

The sampling sites involved in this study are located across Xinjiang. There are considerable differences in the environment, mode, and scale of donkey breeding between southern Xinjiang and northern Xinjiang, which are divided by the Tianshan Mountains. Southern Xinjiang is dominated by desert terrain with a dry climate and little rain, while northern Xinjiang has grassland terrain with a humid climate and abundant rain.

### DNA extraction and PCR amplification

The genomic DNA of each fecal sample was extracted using a commercial E.Z.N. Stool DNA kit (Omega Bio-Tek Inc., Norcross, GA, USA), strictly following the specifications of the manufacturer. All the extracted DNA samples were stored at −20 °C.

*Giardia* was initially screened via nested PCR amplification targeting the *SSU* rRNA gene [1], and further MLG analysis was performed based on the β-giardin (*bg*) [3], glutamate dehydrogenase (*gdh*) [3] and triose phosphate isomerase (*tpi*) genes [19] (Table 1). The target bands of the *SSU* rRNA, *bg*, *gdh* and *tpi* genes were 292 bp, 511 bp, 520 bp, and 530 bp, respectively. Positive (bovine origin *Giardia* assemblage E) and negative (double-distilled water) controls were included in each batch of PCR amplifications.

**Table 1:** Primer sequences and reaction conditions used in nested PCR amplifications.

Target Gene	Primer sequences (5′–3′)	Annealing	Reference
*SSU* rRNA	Gia2029:AAGTGTGGTGCAGACGGACTC	55 °C	[4]
	Gia2150c:CTGCTGCCGTCCTTGGATGT		
	RH11:CATCCGGTCGATCCTGCC	59 °C	
	RH4:AGTCGAACCCTGATTCTCCGCCCAGG		
*tpi*	AL3543:AAATIATGCCTGCTCGTCG	50 °C	[19]
	AL3546:CAAACCTTITCCGCAAACC		
	AL3544:CCCTTCATCGGIGGTAACTT	50 °C	
	AL3545:GTGGCCACCACICCCGTGCC		
*gdh*	GDH1:TTCCGTRTYCAGTACAACTC	50 °C	[6]
	GDH2:ACCTCGTTCTGRGTGGCGCA		
	GDH3:ATGACYGAGCTYCAGAGGCACGT	50 °C	
	GDH4:GTGGCGCARGGCATGATGCA		
*bg*	G7:AAGCCCGACGACCTCACCCGCAGTGC	58 °C	[18]
	G759:GAGGCCGCCCTGGATCTTCGAGACGAC		
	2005F:GAACGAACGAGATCGAGGTCCG	55 °C	
	2005R:CTCGACGAGCTTCGTGTT		

### Sequence analysis

The positive nested PCR amplicons were sent to a commercial sequencing company (GENEWIZ, Suzhou, China) and sequenced on an ABI PRISM™ 3730 XL DNA Analyzer using a BigDye Terminator v3.1 Cycle Sequencing Kit (Applied Biosystems, Foster City, CA, USA). The sequence accuracy was confirmed with bidirectional sequencing, and the sequences were aligned using ClustalX 2.1 (http://www.clustal.org/). Phylogenetic analysis was conducted using the maximum composite likelihood model, and bootstrap values were calculated by analyzing 1,000 replicates and the other chosen default parameters in MEGA 7.0 software (http://www.megasoftware.net/).

### Statistical analysis

A chi-square test was performed, and 95% confidence intervals (CIs) were calculated using Crosstab in SPSS, version 24.0 (SPSS Inc., Chicago, IL, USA). Pearson’s chi-squared test was used for comparisons between two groups, and *p* < 0.05 was considered statistically significant.

### Nucleotide sequence accession numbers

The representative nucleotide sequences were submitted to GenBank at the National Center for Biotechnology Information under accession numbers *tpi* (OQ947877–OQ947879), *gdh* (OQ947880–OQ947881), and *bg* (OQ947882–OQ947885).

## Results

### Occurrence of *Giardia* in donkeys

A total of 129 (17.0%) *Giardia*-positive fecal samples were identified by nested PCR amplification based on the *SSU* rRNA gene. Among the 18 sampling sites, 14 were positive for *Giardia*, with the highest *Giardia* infection rate being 36.6% (15/41) in donkeys in Pishan. The infection rates of *Giardia* in donkeys were statistically significant at different sampling sites (*p* = 0.000) (Table 2).

**Table 2:** Prevalence and distribution of assemblages of *G. duodenalis* infecting donkeys based on amplification and sequence analyses of the *SSU* rRNA gene in Xinjiang, China.

Sampling site	Feeding model	No. samples	No. positive	Infection rate (%)	χ^2^/*p* values/df	Assemblage (*n*)
Qitai	Scale farming	16	0	0.0		–
Bohu		79	20	25.3		B (19), E (1)
Huocheng		20	5	25.0		B (5)
Pishan-1		41	15	36.6		B (15)
Yopurga		103	25	24.3		A (11), B (13), E (1)
Alar		54	17	31.5		A (2), B (15)
Khorgas		20	1	5.0		B (1)
Turpan		17	6	35.3		B (6)
Gongliu		21	3	14.3		B (1), E (2)
Karakax		88	26	29.5		A (5), B (21)
Barkol-1		11	0	0.0		–
Yuli		32	2	6.3		B (2)
Kuqa		78	4	5.1		A (3), B (1)
Sub total	–	580	124	21.4	43.542/0.000/12	A (21), B (99), E (4)
Akqi	Domestic rearing	11	1	9.1		B (1)
Barkol-2		21	0	0.0		–
Zepu		34	1	2.9		B (1)
Yecheng		64	3	4.7		A (2), B (1)
Pishan-2		48	0	0.0		–
Sub total	–	178	5	2.8	4.414/0.353/4	A (2), B (3)
Total	–	758	129	17.0	85.937/0.000/17	A (23), B (102), E (4)

The infection rate in large-scale farm donkeys was 21.4% (124/580), which was significantly higher than that in domestic donkeys (2.8%, 5/178) (*p* = 0.000). The infection rate in <1-year-old donkeys was 19.3% (72/374), which was significantly higher than that in >1-year-old donkeys (12.7%, 41/323) (*p* = 0.019), while the infection rate in donkeys of unclear age was 26.2% (16/61) (Table 3).

**Table 3:** Prevalence and distribution of assemblages of *G. duodenalis* by breeding pattern and age.

Feeding pattern/Age	No. samples	No. positive	Infection rate (%)	χ^2^/*p* values/OR/95% CI	Assemblage (*n*)
Domestic rearing	178	5	2.8	Reference	A (2), B (3)
Large-scale farming	580	124	21.4	33.259/0.000/0.106/0.043–0.264	A (21), B (99), E (4)
<1 years	374	72	19.3	5.487/0.019/1.640/1.081–2.487	A (11), B (57), E (4)
>1 years	323	41	12.7	Reference	A (12), B (29)
Unclear	61	16	26.2		B (16)

### Sequence analysis of *Giardia* in donkeys

Among 129 *Giardia-*positive samples at the *SSU* rRNA gene, 23 samples were identified as *Giardia* assemblage A, 102 samples were identified as assemblage B, and the other 4 samples were identified as assemblage E. Assemblage B was the dominant assemblage in all *Giardia*-positive areas. Assemblage A was found in six areas, and assemblage E was found on only three large-scale farms (Tables 2 and 3). Assemblage B was the common assemblage identified in <1-year-old and >-year-old donkeys, and assemblage E was found only in <1-year-old donkeys (Table 3).

### MLG analysis of *Giardia* isolates from donkeys

For the 129 *Giardia*-positive isolates, multilocus genotyping analysis was performed, and a total of 40, 34, and 59 sequences were obtained at the *bg*, *gdh*, and *tpi* genes, respectively. At the *bg* gene, three assemblage AI sequences and two assemblage B sequences were obtained, including two novel assemblage AI sequences and two novel assemblage B sequences. At the *gdh* gene, two assemblage AI sequences and three assemblage B sequences were obtained, including two novel assemblage B sequences. At the *tpi* gene, three assemblage AI sequences (including a novel sequence) and two assemblage B sequences (including a novel sequence) were obtained (Table 4).

**Table 4:** Assemblage substitutions in *bg*, *gdh*, and *tpi* sequences within *G. duodenalis* assemblage A and assemblage B.

Sequence (*n*)	Nucleotide positions				GenBank ID
*bg*-A	68	112	221	317	
Ref. sequence	T	A	T	T	MK610391
AI-1 (10)	–	–	–	–	
AI-2 a (1)	–	T	C	–	
AI-3 a (6)	C	–	–	C	
*bg*-B	44	1563			MG736242
Ref. sequence	T	G			
B-1 a (1)	C	A			
B-2 a (22)	C	G			
*gdh*-A	168				
ref	T				MN047217
AI-1 (6)	–				
AI-2 (1)	C				MK645799
*gdh*-B	101	327	352	442	
Ref. sequence	A	C	G	G	MK962824
B-1 a (1)	–	T	A	A	
B-2 a (1)	G	T	A	–	
B-3 (25)	–	T	A	–	MN174851
*tpi*-A	114	153	222	384	JQ688289
Ref. sequence	T	G	A	C	
AI-1 (8)	–	–	–	–	
AI-2 a (3)	C	–	G	–	
AI-3 a (1)	C	A	G	T	
*tpi*-B	383				KU892520
Ref. sequence	A				
B-1 (42)	–				
B-2^a^ (5)	G				

N-dash (–) indicates that the sequence is the same as the reference sequence.

a Novel sequence.

The *bg*, *gdh*, and *tpi* genes were successfully genotyped in 21 *Giardia* isolates. Four isolates formed four novel assemblage AI MLG sequences that were named MLG-AI-1 (*n* = 1), MLG-AI-2 (*n* = 1), MLG-AI-3 (*n* = 1), and MLG-AI-4 (*n* = 1). Sixteen isolates of *Giardia* formed three novel assemblage B MLG sequences, that were named MLG-B1 (*n* = 1), MLG-B2 (*n* = 14), and MLG-B3 (*n* = 1). Two isolates formed two MLG-mix sequences. (Table 5).

**Table 5:** Multilocus characterization of *G. duodenalis* isolates based on the *bg*, *gdh*, and *tpi* genes.

Sample number	*bg*	*gdh*	*tpi*	MLG
258	AI-1	AI-1	A1-1	MLG-AI-1
288	AI-3	AI-1	A1-1	MLG-AI-2
326	AI-1	AI-1	AI-3	MLG-AI-3
989	AI-3	AI-2	AI-2	MLG-AI-4
18	B-1	B-3	B-1	MLG-B1
22, 24, 32, 33, 41, 68, 115, 252, 316, 321, 328, 333, 345	B-2	B-3	B-1	MLG-B2
210	B-2	B-3	B-2	MLG-B3
330	AI-1	AI-1	B-1	MLG-mix
976	AI-1	B-3	B-1	MLG-mix

### Phylogenetic analysis of *Giardia* isolates

**Figure 2:**
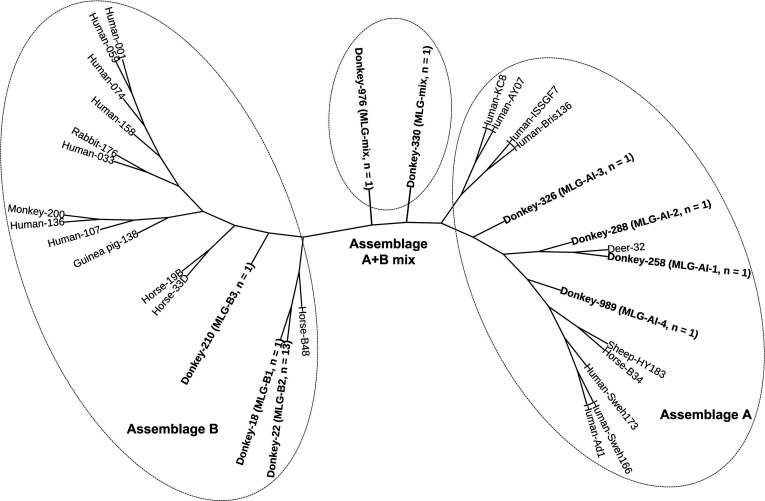
The unrooted tree of phylogenetic relationships among *G. duodenalis* subassemblage (A, B, A and B mixed) MLGs at the tpi, gdh, and bg loci determined with the maximum likelihood method based on the general time reversible model. Bootstrap values >50% derived from 1000 replicates are shown at the nodes. New multilocus sequences determined in the present study are indicated in bold.

A phylogenetic analysis of the concatenated sequences of subassemblages A, B and A mixed with B based on three loci (*bg*, *gdh*, and *tpi*) was performed. Clear host separation was seen among all the samples. The vast majority of the donkey samples were isolated for the single clade, although there were two slightly different cases, one A in the same cluster as deer and the other B in the same cluster as horses (Fig. 2). Compared with the closer genetic distance to human or nonhuman primates shown by other animal hosts, including guinea pigs, sheep, deer and horses, the donkey-derived isolates in this study showed higher genetic uniqueness (Fig. 2).

## Discussion

This study used the same detection methods as two other studies, but has a bigger sample size, and the *Giardia* infection rate was 17.0% (129/758), which was higher than the previous report in the same region (14.8%, 31/210), and some other regions in China, such as Inner Mongolia (6.8%. 12/176), Jilin Province (10.4%. 5/48), Liaoning Province (13.8%, 4/29), and Shandong Province (14.7, 38/258) [11, 24]. The infection rate of *Giardia* in donkeys varies greatly in different regions of China, but the sample size in some regions is small [11, 24]; further research is still needed. Many factors could cause differences in infection rates, such as detection methods, season, region, sampling time, and sample sizes [7].

The results of this study show that the age and feeding style of donkeys were associated with *Giardia* infection rates, with a higher infection rate in young animals. The *Giardia* infection rate in donkeys has been shown to be age-related, and the infection rate of young donkeys is higher [11, 24]. The same phenomenon has been seen in studies of other animals [4, 6, 13]. In this study, the *Giardia* infection rate in large-scale farm donkeys was significantly higher than that in domestic donkeys. There is a lack of studies on the relationship between the prevalence of *Giardia* infection and feeding patterns in donkeys; previous reports have shown that the prevalence of *Giardia* infection is not related to feeding pattern in sheep [7]. At present, studies of *Giardia* in donkeys are still limited around the world. In the future, it will be necessary to further strengthen the molecular epidemiological investigation of *Giardia*.

*Giardia* assemblages A, B and E were identified in this study, and assemblages A and B are two common assemblages, which is consistent with previous reports [11, 24]. Assemblages A and B are distributed in humans as well as several other vertebrate animals [6, 14]. Assemblage E was identified as the most common assemblage in ungulates [16, 22] and has been found in humans [5, 23]. Assemblage E was identified in horses [9, 20], but has rarely been reported in donkeys [11]. Interestingly, assemblage E was found only in young and large-scale farm donkeys, suggesting that infection with assemblage E in donkeys may be associated with age and feeding pattern. In China, assemblages A and B of *Giardia* are common in humans, livestock, companion animals, wild animals, nonhuman primates, and even in wastewater, suggesting that interspecies transmission of *Giardia* may be common in China [7, 12, 17, 21].

The novel sequences of subassemblages A and B obtained at three genes in this study showed that *Giardia* in donkeys may have unique subassemblages A and B, which is in agreement with previously published conclusions [24]. The MLG analysis at three genes showed the presence of four novel MLGs in assemblage AI and three novel MLGs in assemblage B. Assemblage A can be well divided into three subtypes through multiple sites, while assemblage B may have more subtypes [6]. Meanwhile, previous studies have shown that mixed infections could be detected by MLG analysis [2, 3], and two cases of assemblages A and B mixed infection were found in this study. Phylogenetic analysis showed that the assemblage A isolates are closer to those from deer than to those from humans, and assemblage B isolates show a higher degree of genetic similarity than other isolates, indicating that *Giardia* in donkeys may have unique evolutionary characteristics.

## Conclusion

The results showed that *Giardia* is a common parasite in donkeys in Xinjiang. Young donkeys are more susceptible to *Giardia*, with assemblage B being the predominant assemblage. The MLG results showed that *Giardia* assemblages A and B from donkeys have genetic diversity and host specificity.

## Conflicts of interest

The authors declare that there are no conflicts of interest.
